# NCAPH Promotes the Proliferation of Prostate Cancer Cells Via Modulating the E2F1 Mediated PI3K/AKT/mTOR Axis

**DOI:** 10.7150/ijms.103444

**Published:** 2025-01-27

**Authors:** Qing Shi, Jinpeng Ma, Xiang Pan, Te Liu, Kailai Chen, Meiqi Xu, Zhichong Wu, Bin Sun, Manjie Zhang, Yakun Luo

**Affiliations:** 1 NHC Key Laboratory of Molecular Probes and Targeted Diagnosis and Therapy, Harbin Medical University, Harbin 150001, China.; 2 Research Center for Pharmacoinformatics, College of Pharmacy, Harbin Medical University, Harbin 150081, China.; 3 Department of General Surgery, Ruijin Hospital, Shanghai Jiao Tong University School of Medicine, Shanghai 200025, China.; 4 Department of Urology, The Fourth Affiliated Hospital of Harbin Medical University, Harbin 150001, China.

**Keywords:** prostate cancer, NCAPH, E2F1, PI3K/AKT/mTOR, therapeutic strategy

## Abstract

Prostate cancer (PCa) remains a major challenge in oncology, driving the need for continuous exploration and development of innovative treatment strategies. NCAPH plays a critical role in tumorigenesis and progression across multiple cancer types; however, its specific role in PCa has yet to be fully understood. This study aims to elucidate the biological functions of NCAPH in PCa. Our findings reveal that *NCAPH* gene expression is upregulated in PCa patients and correlates with poor prognosis. Enrichment analysis, flow cytometry, and correlation analysis demonstrate that NCAPH promotes the PI3K/AKT/mTOR pathway and facilitates cell cycle transition in PCa cells. Additionally, we identified E2F1 as a novel downstream target of NCAPH in PCa cells. Mechanistically, ChIP analysis showed that NCAPH regulates *E2F1* transcription by binding to the proximal promoter of *E2F1*, subsequently stimulating the PI3K/AKT/mTOR pathway and activating downstream targets for cell cycle progression in PCa cells. Notably, combining *NCAPH* knockdown with an mTOR inhibitor (Everolimus) or a cyclin-dependent kinase inhibitor (Flavopiridol) demonstrated promising anti-tumor effects both *in vitro* and *in vivo*. This study highlights the significant pro-tumor role of NCAPH in PCa and suggests its potential as a therapeutic target.

## Introduction

Prostate cancer (PCa) is a major health problem affecting men worldwide. It is the second most common cancer in men and the fifth leading cause of cancer-related death worldwide 1,2. Despite advances in diagnostic techniques and treatment modalities, the molecular processes driving PCa progression remain poorly understood. It is imperative to identify key genes and pathways associated with PCa cell proliferation to pave the way for targeted therapeutic intervention and improved patient outcomes 3,4.

The *NCAPH* gene, also known as non-SMC condensin I complex subunit H, encodes the NCAPH protein, which plays a key role in chromosome condensation and organization during cell division 5,6. It is a component of the condensin I complex and is essential for ensuring proper chromosome segregation during mitosis and meiosis 7. Aberrant expression of the *NCAPH* gene has been found in multiple cancer types, including breast 8, lung 9, and bladder cancer 10. Notably, in cervical cancer, upregulated NCAPH binds to human papillomavirus E7 (HPV E7) and enhances cell proliferation by activating the PI3K/AKT/SGK pathway 11.

In colon cancer, *NCAPH* depletion inhibits tumor growth and migration and induces apoptosis and cell cycle arrest 12. Additionally, NCAPH plays a role in gastric cancer progression by regulating the DNA damage response 13. In prostate cancer, Arai et al. reported that NCAPH upregulation is associated with increased invasion and migration of PCa cells 14. However, the specific mechanism through which NCAPH influences PCa cell malignancy remains unclear.

Our study demonstrates that high *NCAPH* expression is positively correlated with poor prognosis in PCa patients. We show that NCAPH promotes cell cycle progression in PCa cells by activating the E2F1-mediated PI3K/AKT/mTOR pathway *in vitro*. Furthermore, *NCAPH* gene silencing (NCAPH-KD) significantly inhibits tumor growth in a xenograft nude mouse model of PCa *in vivo*. Combining NCAPH-KD with an mTOR inhibitor (Everolimus, Eve) or a cyclin-dependent kinase inhibitor (Flavopiridol, Flav) shows a promising anti-tumor effect in PCa cells both *in vitro* and* in vivo*. These findings suggest that NCAPH plays a pro-tumor role in PCa and could serve as a potential therapeutic target.

## Materials and Methods

### Patients and specimens

Tissue specimens were procured from patients undergoing surgical procedures at the Department of Urology, Fourth Hospital of Harbin Medical University, spanning the period from January 2022 to January 2023, were diagnosed with prostate cancer. Ethical clearance for all investigations involving human specimens was obtained from the Ethics Committee of Fourth Affiliated Hospital of Harbin Medical University (2021-WZYSLLSC-31). The study was conducted in accordance with the guidelines in the Declaration of Helsinki.

### Cell culture

The utilized cell lines encompassed human normal prostate cells (RWPE-1) along with three distinct prostate cancer cell lines (LNCaP, PC3, and DU145), all procured from ATCC. LNCaP and PC3 cell lines were propagated in RPMI 1640 medium (A1049101, Gibco, USA), while the DU145 cell line was maintained in DMEM medium (21013024, Gibco, USA). The RWPE-1 cell line was cultured in K-SFM medium (17005042, Invitrogen, USA). Each medium variant was supplemented with 10% heat-inactivated fetal bovine serum (16140071, Gibco, USA) and 1% penicillin-streptomycin (C0222, Beyotime, China). The cell lines were maintained in an incubator with a humidified atmosphere of 5% CO_2_ at 37 °C. Additionally, PCR testing validated the absence of mycoplasma contamination in all cell lines.

### RNA interfering and transfection

The siRNAs utilized comprised of siNCAPH#1 (siG000029781A-1-5), siNCAPH#2 (siG000029781B-1-5), siE2F1#1 (siG14101792341-1-5), siE2F1#2 (siG14101792354-1-5), and the negative control siRNA (siCtrl, siN00000001), all sourced from Ribo Biotechnology Company (Guangzhou, China). Transient transfection of the cells was facilitated using Lipofectamine 2000 (11668019, Invitrogen, USA). Subsequently, protein and RNA were extracted to validate the efficacy of the knockout.

### RNA extraction, cDNA synthesis, and real-time PCR analysis

Total RNA from both tissues and cells was extracted using the Trizol reagent, with subsequent quantification accomplished via the NanoDrop 2000 spectrophotometer. Reverse transcription of RNA utilized the Reverse Ace qPCR RT Master Mix kit, specifically designed for messenger RNA (mRNA). Real-time quantitative PCR (RT-qPCR) assays were conducted on the StepOne Plus Real-time PCR system employing the TaqMan Gene Expression Assay kit. Gene expression levels were evaluated utilizing the 2^-ΔΔCT^ method, with β-actin employed as the internal reference gene. Each RT-qPCR experiment set was performed in triplicate and independently replicated three times. The primers utilized are detailed in Supplementary Table S1.

### Transwell assay

The cell migration assay was conducted by seeding cells onto the upper surface of a transwell chamber. Following a 24-hour incubation period, the invaded cells were fixed using 4% paraformaldehyde and subsequently stained with 0.5% crystal violet. The invasive cell populations were photographed by microscope and counted by ImageJ.

### Cell proliferation assay

CCK-8 analysis (Cell Counting Kit-8, HY-K0301, MCE) was employed to assess cell proliferation. Cells were seeded into wells of a 96-well plate at a density of 3,000 cells per well. Following inoculation, cells were subjected to continuous incubation for 0, 24, 48, 72, and 96 hours post-treatment. Subsequently, CCK-8 reagent was introduced into each well, followed by a 2 hours incubation period at 37 °C, after which absorbance measurements were taken to determine cell viability.

### Cell colony formation assay

In colony formation assay, cells were plated in 6-well culture plates at a density of 5×10^2^ cells per well for prostate cancer cells and cultured for 2 weeks. After the incubation period, colonies were stained with 0.5% crystal violet. Plate images were subsequently analyzed utilizing ImageJ software. Each experiment was conducted independently in triplicate, and statistical analyses were conducted using Prism software.

### Protein extraction and western blotting

Cell lysates were obtained by incubating cells for 30 minutes on ice with RIPA lysis buffer. Subsequently, the cell lysates were centrifuged at 12,000 g for 15 minutes at 4 °C. Protein concentration in the lysates was determined using the BCA Protein Assay kit. The proteins were then separated by SDS-PAGE and transferred to PVDF membranes. The membranes were blocked with 5% BSA in PBS and incubated with primary antibodies overnight at 4 °C. The next day, the membranes were incubated with the corresponding secondary antibody for 2 hours at 25 °C. Protein bands were visualized using chemiluminescence substrates. Details of the antibodies used are provided in Supplementary Table S2.

### Flow cytometric assay

Cell cycle and apoptosis analysis were performed by flow cytometry. For cell cycle analysis, cells were immobilized with 70% ethanol at 4°C for 2 hours and then incubated with propyl iodide (PI) and RNase A (C1052, Beyotime) for 30 minutes. For apoptosis analysis, cells were incubated with Annexin V-FITC and PI (C1062, Beyotime) at room temperature for 30 minutes. Subsequently, cell cycle and apoptotic rates were measured using flow cytometry (EPICS, Xl-4; Beckman Coulter, Inc., Brea, CA, USA).

### Co-immunoprecipitation (Co-IP) Assay

The Co-IP assay was performed according to the manufacturer's protocol as previously described. Briefly, cellular lysis was conducted, and protein extraction was carried out using Cell Lysis Buffer for Immunoprecipitation (#9803, Cell Signaling Technology). Anti-NCAPH (11515-1-AP, Proteintech) or anti-E2F1 (66515-1-Ig, Proteintech) antibodies were utilized for the pull-down assay, which was conducted overnight at 4°C with gentle agitation at 30 rpm. Following this, Protein A + G Agarose (20423, Thermo Scientific) was added and gently mixed at 4 °C for 2 hours at 20 rpm. The resultant precipitate was then centrifuged, washed, and combined with SDS-PAGE sample loading buffer (#3401, Merck). Denaturation was achieved by heating at 100°C for 15 minutes. Rabbit-purified IgG was employed as the negative control.

### Chromatin immunoprecipitation assay

The Chromatin Immunoprecipitation (ChIP) assay was conducted following the previous manufacturer protocol 15. Initially, a total of 2×10^7^ cells were processed using the ChIP assay kit (17-295, Millipore, Massachusetts, USA) in accordance with the manufacturer's instructions. The cross-linked chromatin underwent sonication to generate fragments ranging from 200 to 500 base pairs in length. Subsequently, the chromatin was subjected to immunoprecipitation using antibodies specific to NCAPH (11515-1-AP, Proteintech), E2F1 (66515-1-Ig, Proteintech), CDK1 (19532-1-AP, Proteintech) or CyclinB1 (55004-1-AP, Proteintech). The resulting DNA fragments were purified using the QIAquick PCR purification kit (28104, Qiagen, Hilden, Germany) and employed for RT-qPCR reactions with iTaq Universal SYBR Green (1725124, Bio-Rad). Each ChIP assay was independently repeated at least three times. Details regarding the primers used for ChIP-qPCR can be found in Supplementary Table S1.

### Immunohistochemistry (IHC) assay

Paraffin-embedded human PCa tumor tissues and adjacent tissues and xenograft tumor tissues from nude mice were processed for the protein analysis. Specifically, the tissue slices were cut to a thickness of 4 μm, spread at 42 °C water temperature, and subsequently baked in a 60 °C oven for 30 minutes. Following dewaxing in xylene solution, the sections were rehydrated using different alcohol concentrations (100%, 90%, 80%, 75%). Subsequently, they were blocked with 1% BSA and incubated overnight with primary antibody at 4 °C. The secondary antibody was applied at room temperature for 2 hours. The signals were detected with DAB (DA1010, Solarbio). Finally, nuclear staining was performed, followed by dehydration and sealing with a neutral adhesive, enabling observation of tissue staining under a microscope.

### Hematoxylin-eosin (HE) staining assay

HE staining assays were conducted utilizing the HE staining kit (G1120, Solarbio, China), following the supplier's guidelines. Briefly, after the samples underwent deparaffinization and hydration, they were subjected to hematoxylin staining for 2 minutes, followed by rinsing with PBS. Subsequently, eosin staining was performed for 1 minute, followed by PBS washing for 5 minutes. Finally, the samples were dehydrated, permeated, sealed with neutral resin, dried, and photographed under a microscope.

### Molecular docking

The AlphaFold-predicted protein structures of NCAPH and E2F1 were obtained from the UniProt database, with access codes O00255 and Q08050, respectively. These structures underwent 50 ns production molecular dynamics (MD) simulations using the Amber 20 package to refine their structures. The Amber ff19SB force field was employed for the MD simulations. The final frame of the MD trajectory refinement was extracted to serve as input structures for protein-protein docking. Free protein-protein docking was carried out using the online ZDOCK server without specifying any contacting constraints. The top ten highest-ranked docking poses were selected for further analyses. Structure renderings were generated using the ChimeraX software.

### Xenograft tumor model

Athymic nude BALB/c male mice aged 6 weeks were procured from Beijing Vital River Laboratory Animal Technology Co., China. The mice were randomly assigned to 7 groups, each containing 5 mice. LNCaP cells (1×10^7^ cells/mice) were suspended in a 200 μL mixture of 50% PBS and 50% Matrigel (BD Biosciences) and injected subcutaneously into the right lower limb back of the mice to establish a xenograft model. After 15 days of acclimatization, when the subcutaneous tumors reached a size of 50 mm^3^, various treatment methods were initiated. Tumor size was monitored using vernier calipers every week following the initial injection of siCtrl, siNCAPH, DMSO, Everolimus (20 nM/mice), or Flavopiridol (100 nM/mice) as indicated. Tumor volumes were determined using the following formula: (length × width^2^)/2. After 42 days, the nude mice were euthanized, and the subcutaneous tumors were excised, photographed, weighed, and immediately fixed in 4% paraformaldehyde for further analysis. All animal studies were conducted in accordance with the National Institute of Health guidelines for the Care and Use of Laboratory Animals and approved by the Ethics Committee of Harbin Medical University.

### Data mining analysis

Gene expression data (measured in transcripts per million, TPM) and relevant prostate cancer prognostic and clinical data were sourced from The Cancer Genome Atlas (TCGA) databases (https://portal.gdc.cancer.gov/). GSVA enrichment analysis was conducted using the "GSVA" R package (23323831). The identification of differentially expressed genes (DEGs) was performed using the “limma” package (25605792). DEGs were selected based on an adjusted p-value less than 0.05 and an absolute value of log2 Fold Change greater than or equal to 0.1. Pearson correlation analysis was employed for assessing correlations.

### Statistical analyses

All statistical analyses were conducted using GraphPad Prism 8.0 (GraphPad Software Inc., San Diego, CA, USA), and data were presented as mean ± standard deviation (SD). Differences between two groups were assessed using Student's t-test, while comparisons among three or more groups were initially evaluated using one-way analysis of variance (ANOVA). Cut-off values for survival analysis were determined using the Maxstat (R package). Results with a p-value of 0.05 or less were deemed statistically significant.

## Results

### NCAPH plays an oncogenic role in PCa

**Figure 1:**
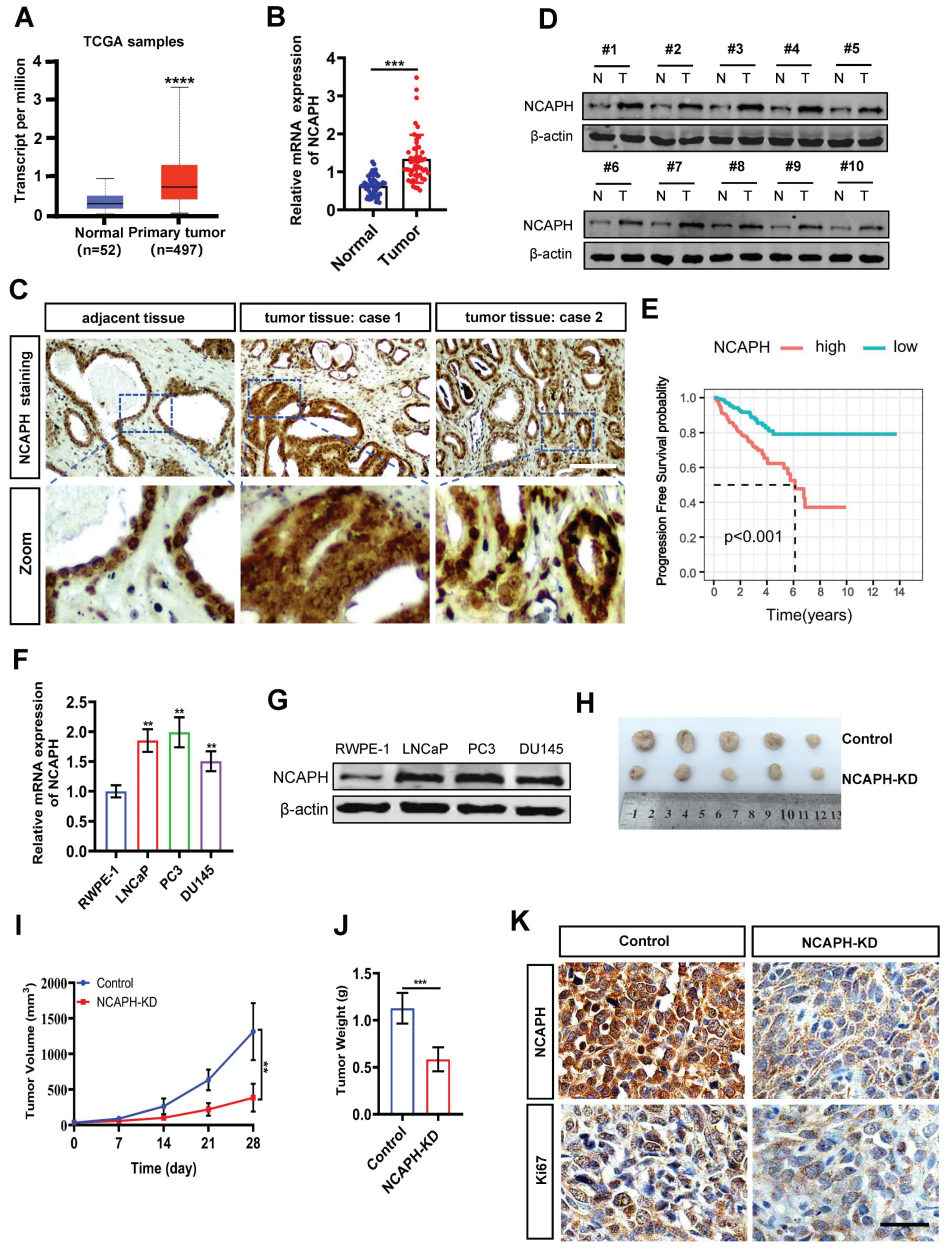
** The expression of NCAPH is considered to be upregulated in prostate cancer tissues and prostate cancer cells.** (A) The expression status of the *NCAPH* gene in PCa based on TCGA database. (B) *NCAPH* mRNA expression was upregulated in PCa compared with adjacent normal tissues. (C and D) NCAPH protein expression was upregulated in PCa tumor tissues than that in adjacent normal tissues as determined by IHC and western bloting. (E) The survival analysis indicated that the *NCAPH* gene was a risk factor and was related to the survival and prognosis of PCa tissues based on GEO database. (F and G) The mRNA and protein levels of NCAPH in PCa cell lines are determined by RT-qPCR and western bloting. (H) Images of representative nude mouse xenograft model tumor. LNCaP tumor xenografts excised from male BALB/c (nu/nu) nude mice after 28 days treatments with the* in vivo* RNA interfering of knockdown the *NCAPH* gene (NCAPH-KD group) or control group. (I and J) Days versus tumor volume curves and tumor weight for LNCaP tumor xenografts show that NCAPH-KD inhibited tumor growth compared with control group. (K) Representative IHC staining images of NCAPH and Ki67 were presented in NCAPH-KD-treated or control-treated LNCaP xenograft nude mice tissues. Scale bar, 50 µm. ns, non-significant, * P < 0.05, ** P < 0.01, *** P < 0.001, **** P < 0.0001 vs control.

To determine the role of the *NCAPH* gene in PCa, we first analyzed *NCAPH* mRNA levels in human PCa tissues using data from The Cancer Genome Atlas (TCGA). As shown in Figure 1A, *NCAPH* was significantly upregulated in PCa tissues (n=497) compared to normal tissues (n=52). We further examined *NCAPH* expression in 24 pairs of clinical prostate cancer tissues (tumor tissues, n=24; adjacent tissues, n=24) using real-time quantitative PCR (RT-qPCR) and immunohistochemistry (IHC). Compared to adjacent tissues (n=24), *NCAPH* mRNA was significantly elevated in PCa tissues (Figure 1B). The IHC results also showed upregulated NCAPH expression in clinical PCa tissues, with representative IHC images displayed in Figure 1C. To confirm these findings, we assessed NCAPH expression in 10 pairs of PCa tissues using western blot analysis. Consistent with the IHC results, NCAPH levels were higher in prostate tumor tissues than in adjacent tissues (Figure 1D). Importantly, we found that the overall survival (OS) rate of patients with high NCAPH expression was significantly lower than that of patients with low NCAPH expression, based on TCGA data (Figure 1E). We then investigated the function of NCAPH in PCa cells and found a notable upregulation of NCAPH at both mRNA and protein levels in the PCa cell lines LNCaP, PC3 and DU145 compared to normal human prostate epithelial cells (RWPE-1) (Figure 1F and G). Moreover, *In vivo* studies using the LNCaP-xenografted nude mouse model showed that *NCAPH* knockdown (NCAPH-KD) via RNA interference significantly reduced the size of xenograft tumors (Figure 1H-J). We also observed a substantial decrease in NCAPH and Ki67 expression in NCAPH-KD xenograft tumor tissues (Figure 1K). These findings suggest that NCAPH acts as an oncogenic factor in PCa cells both* in vitro* and *in vivo*.

### NCAPH-KD inhibits cell proliferation and induces apoptosis in PCa cells

**Figure 2:**
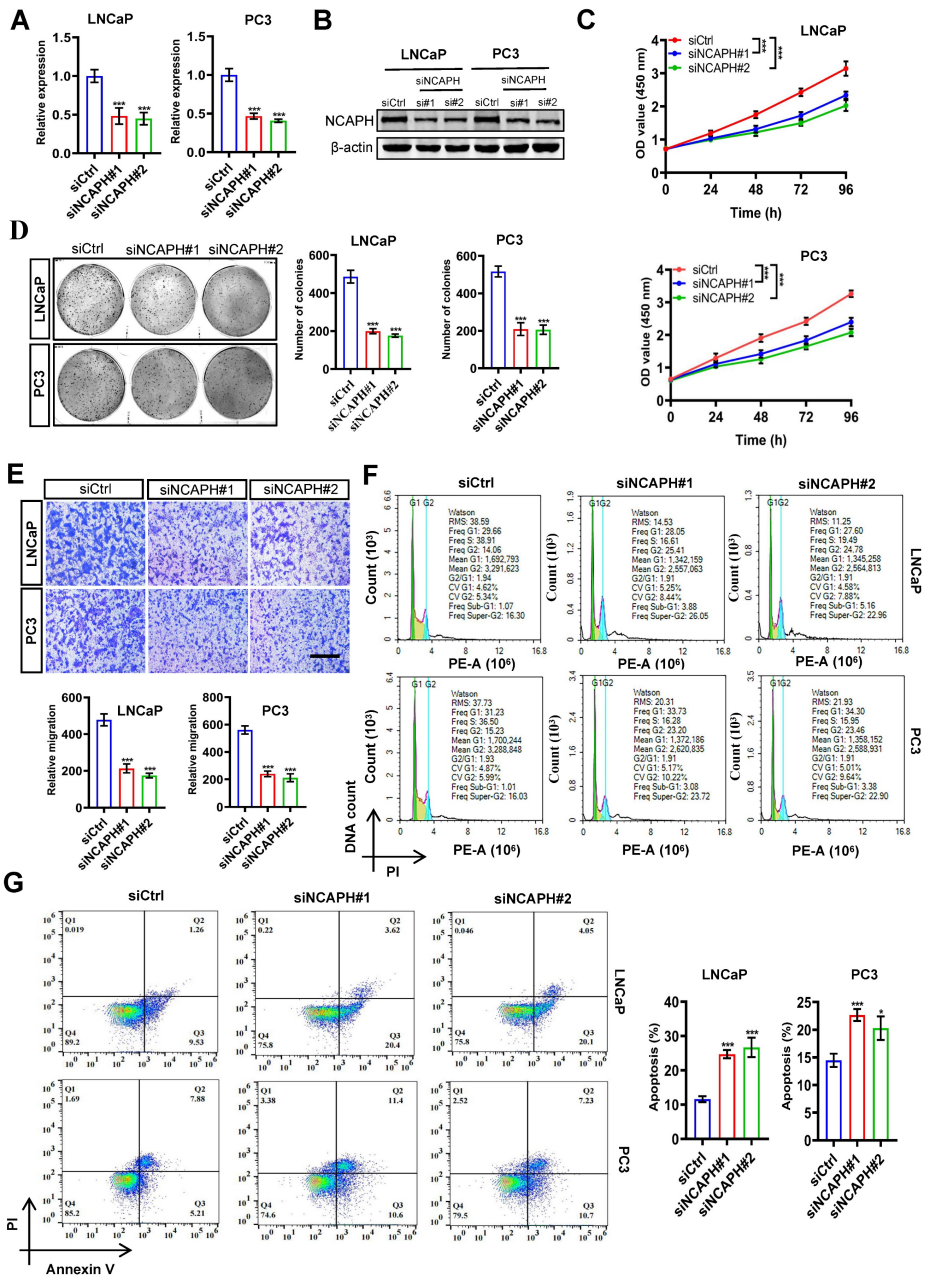
***NCAPH* depletion inhibits the maligant potentials of PCa cells.** (A) RT-qPCR analysis of *NCAPH* transcription in siCtrl-, siNCAPH#1- or siNCAPH#2-treated LNCaP and PC3 PCa cells. Data were normalized against *ACTB* and represented as fold change. (B) Western blotting showing NCAPH expression in siCtrl-, siNCAPH#1- or siNCAPH#2-treated LNCaP and PC3 PCa cells. (C) CCK-8 analysis showing the effect of NCAPH-KD (siNCAPH#1, siNCAPH#2) on the cell proliferation of the tested PCa cell lines. (D) Representative images of colony formation assays and their quantification in NCAPH-KD LNCaP and PC3 PCa cells. (E) NCAPH-KD in LNCaP and PC3 cells significantly inhibited cell migrational capacity. (F) NCAPH-KD with siNCAPH#1 or siNCAPH#2 induced cell cycle arrest at the G2/M phase in LNCaP and PC3 PCa cells, compared to the cells transfected with siCtrl-by flow cytometry. (G) NCAPH-KD with siNCAPH#1 or siNCAPH#2 induced cell apoptosis in LNCaP and PC3 PCa cells. Scale bar, 100 µm. ns, non-significant, * P < 0.05, ** P < 0.01, *** P < 0.001 vs control.

To further evaluate the function of the *NCAPH* gene in PCa cells, we conducted RNA interference (siNCAPH: siNCAPH#1 or siNCAPH#2) to induce *NCAPH* knockdown (NCAPH-KD) in PCa cells, using non-targeting siRNA as a negative control (siCtrl). The efficiency of NCAPH-KD in PCa cells was assessed by RT-qPCR and western blotting. As shown in Figure 2A and B, both *NCAPH* gene expression and NCAPH protein levels were significantly reduced in NCAPH-KD LNCaP and PC3 cells compared to siCtrl-treated cells at 72 hours post-transfection. To assess cell proliferation, we then performed CCK-8 and colony formation assays in NCAPH-KD and siCtrl-treated LNCaP and PC3 cells. The results showed that proliferation of LNCaP and PC3 cells was significantly inhibited following NCAPH-KD treatment (Figure 2C and D). Next, we performed transwell analysis to examine whether NCAPH affects the migrational capacity of PCa cells. Our findings revealed that NCAPH-KD significantly inhibited the migration of LNCaP and PC3 cells (Figure 2E). Furthermore, we confirmed that NCAPH-KD induced cell cycle arrest at the G2/M phase in PCa cells (Figure 2F). In addition, we found that NCAPH-KD induced increased apoptosis in LNCaP and PC3 cells (Figure 2G). Overall, our current results indicated that NCAPH is a key factor regulating PCa cell proliferation and apoptosis.

### NCAPH promotes the proliferation of PCa cells via modulating E2F1/PI3K/AKT/mTOR axis

**Figure 3:**
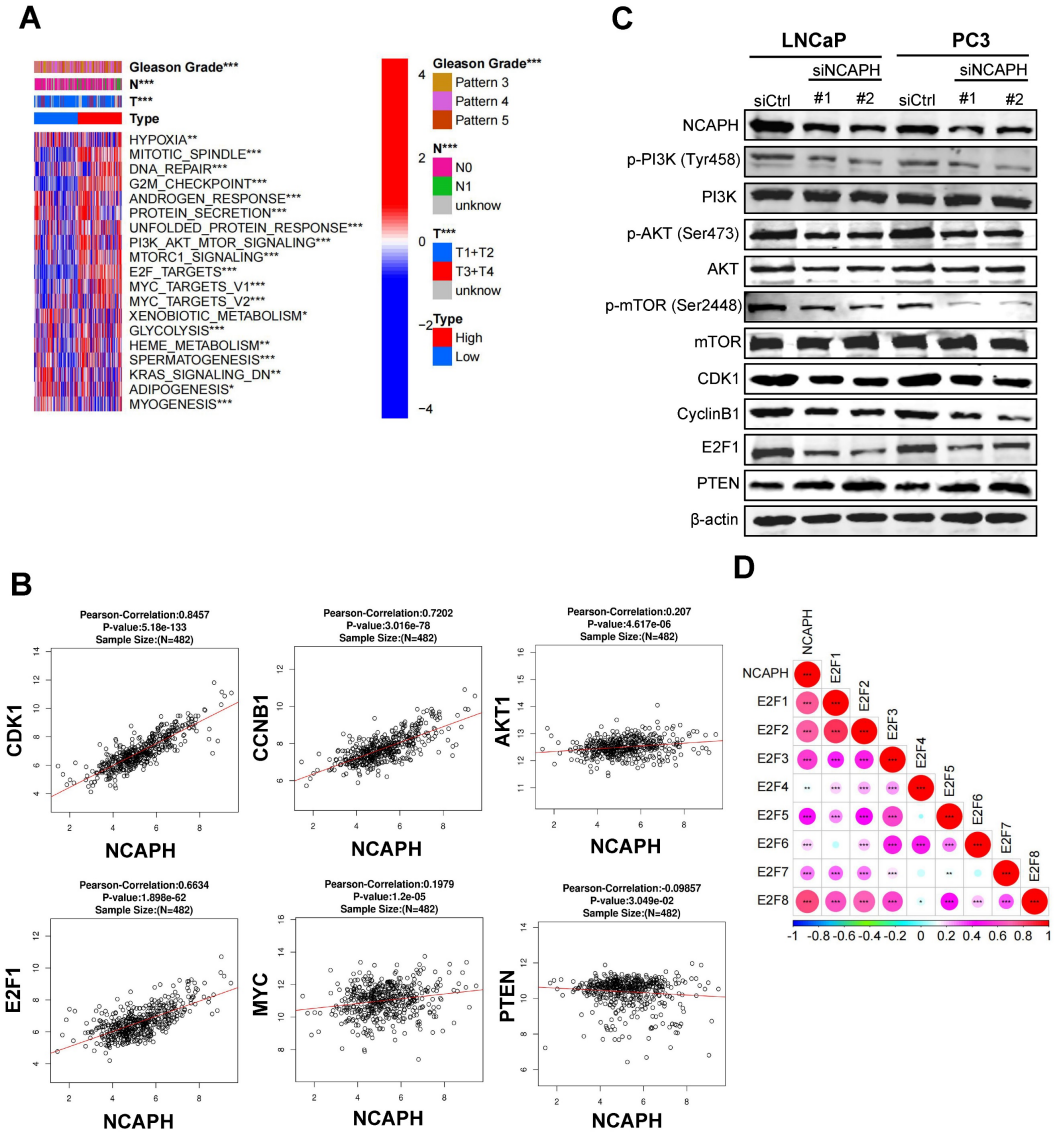
** NCAPH is a vital regulator of PI3K/AKT/mTOR axis.** (A) Differential enrichment pathways and Gleason grades in high - and low-expression of the* NCAPH* gene clinical groups from the TCGA database. (B) The correlation between the *NCAPH* gene and cell cycle related markers, PI3K/AKT/mTOR markers in PCa which based on TCGA database. (C) NCAPH-KD reduced the protein expression of PI3K/AKT/mTOR signaling related markers and cell cycle related markers in LNCaP and PC3 cells. (D) The relationship between *NCAPH* gene and *E2F* family genes was analyzed by TCGA database. ns, non-significant, * P < 0.05, ** P < 0.01, *** P < 0.001 vs control.

To further investigate the mechanism by which the *NCAPH* gene regulates PCa cell proliferation, we next explored differentially enriched pathways in the NCAPH high- and low-expression clinical groups from the TCGA database. Based on GSVA (gene set variation analysis), we found that NCAPH was associated with proliferation pathways such as the E2F1-related pathway, the G2/M checkpoint, and the PI3K/AKT/mTORC1 signaling pathway. At the same time, we noticed that NCAPH was associated with Gleason score, tumor stage, and lymph node metastasis (Figure 3A). We also found a strong correlation between the *NCAPH* gene and several other genes: CDK1 (cor = 0.85), E2F1 (cor = 0.66), CCNB1 (cor = 0.72), PTEN (cor = -0.10), MYC (cor = 0.20), and AKT1 (cor = 0.21) (Figure 3B). These findings suggested that NCAPH is a critical driver of cell proliferation through the activation of the PI3K/AKT/mTORC1 pathway mediated by E2F1 in PCa. Importantly, the transcription factor E2F1 is widely recognized for its role in controlling cell cycle progression 16. Previous studies have revealed that *E2F1* is a key gene in PCa progression 17,18. More intriguingly, E2F1 plays a crucial role in activating the PI3K/AKT/mTOR signaling pathway in hepatocellular carcinoma cells 19. However, the relationship between NCAPH and the E2F1/PI3K/AKT/mTOR axis remains unclear.

Next, we sought to determine whether NCAPH promotes the proliferation of PCa cells by activating the PI3K/AKT/mTOR pathway. In LNCaP and PC3 cells treated with NCAPH-KD, we found that NCAPH-KD reduced the expression levels of E2F1, CDK1, Cyclin B1, phosphorylated PI3K, phosphorylated AKT, and phosphorylated mTOR (Figure 3C). Furthermore, our exploration of the relationships among E2F family genes revealed that E2F1 had the highest correlation with NCAPH, suggesting that *E2F1* may be a downstream target gene of NCAPH (Figure 3D). Our data demonstrate that NCAPH plays an oncogenic role in PCa cells by activating the E2F1/PI3K/AKT/mTOR pathway.

### NCAPH regulates E2F1 transcription in PCa cells

**Figure 4:**
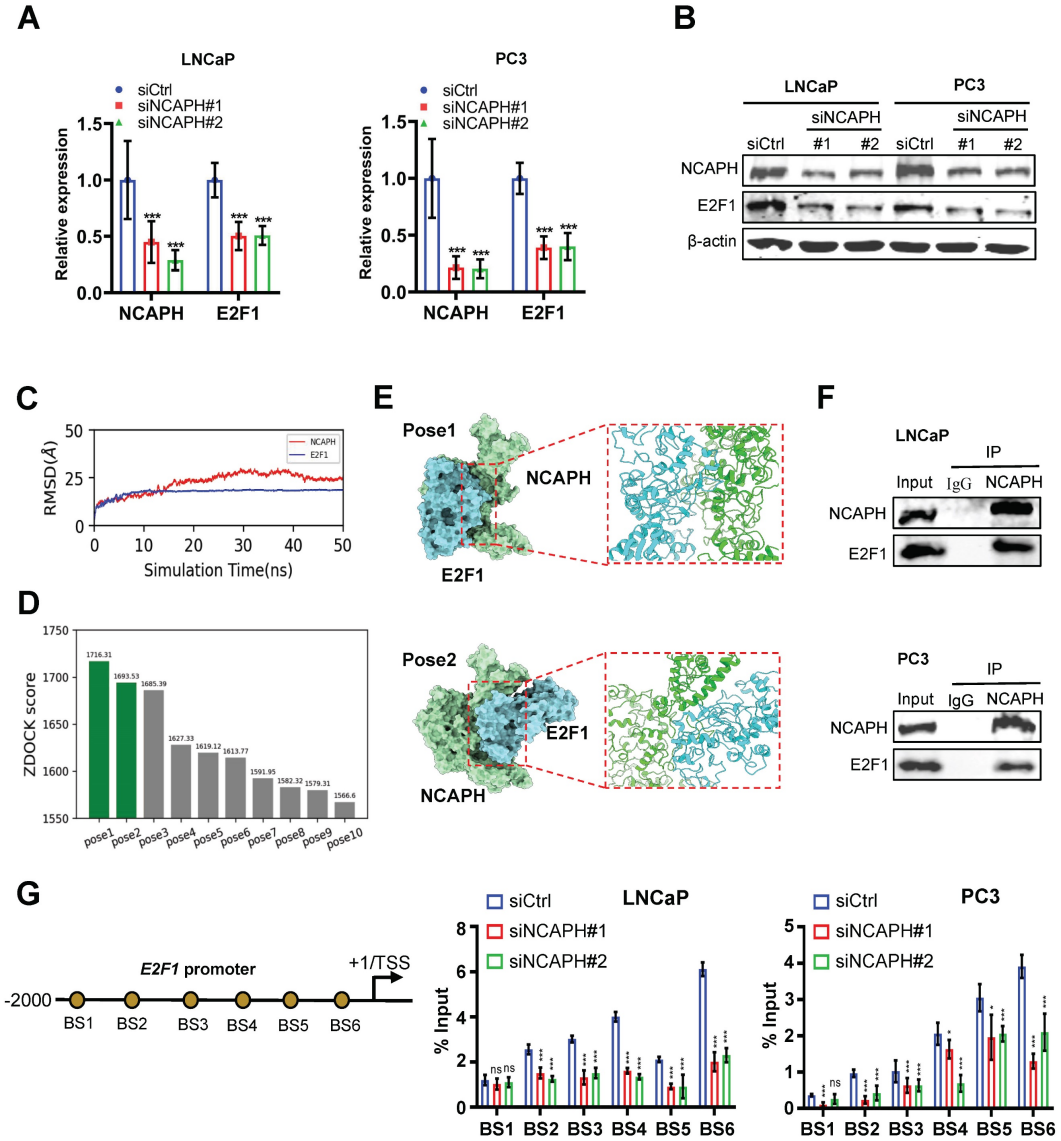
** NCAPH favorably regulates the expression of *E2F1*.** (A) RT-qPCR analysis showing down-regulated E2F1 expression in NCAPH-KD LNCaP and PC3 cells. (B) Western blotting analysis showing NCAPH-KD caused a strong reduction of E2F1 expression in LNCaP and PC3 cells. (C) The RMSD of NCAPH and E2F1 during the 50 ns MD refinement simulations. (D) Histograms of the top 10 ZDOCK binding scores of NCAPH-E2F1 poses. (E) Molecular docking patterns for NCAPH and E2F1. (F) The physical interaction between NCAPH and E2F1 in LNCaP and PC3 cells were detected by Co-IP. (G) Schematic representation of the position of ChIP-qPCR primers along the *E2F1* promoter. ChIP-qPCR analyzing the NCAPH recruitment to the *E2F1* promoter in siNCAPH#1- or siNCAPH#2-treated LNCaP and PC3 cells. ns, non-significant, * P < 0.05, ** P < 0.01, *** P < 0.001 vs control.

To further investigate the mechanism by which NCAPH regulates proliferation through E2F1 in PCa cells, we analyzed E2F1 expression in NCAPH-KD PCa cells using RT-qPCR and Western blotting. Our results showed that NCAPH-KD significantly reduced E2F1 expression at both the mRNA and protein levels in LNCaP and PC3 cells (Figure 4A and B). Next, we explored the interaction between NCAPH and E2F1 in PCa cells. The AlphaFold-predicted structures of NCAPH and E2F1 were obtained from the Uniprot database with access IDs of O00255 and Q08050, respectively. These structures were then subject to 50 ns long implicit solvent MD simulations to refine their structures.

The MD refinement consists of three sequential stages: a. 20000 steps of energy minimization (first 200 steps using the steepest descent algorithm and the rest using the conjugate gradient algorithm). b. Heating the system up to 300 K over 1 ns, with 5 kcal/mol/Å2 harmonic restraints applied on the heavy atoms (no-hydrogen atoms) of the protein. After reaching 300 K, a 50 ns production MD was conducted with all restraints removed. The Amber ff19SB force field was used 20, and the non-bonded interaction cutoff was 10 Å, and the time step was 2 fs. The MD simulations were performed via the Amber20 package 21. For the 50 ns long MD refinement simulations, we calculated the RMSD (root mean square deviation) relative to the starting structure to justify if the structures have become stable. As shown in Figure 4C, the RMSD curves of both proteins became plateaued, meaning that the structures are stable. For each protein, the last frame of the 50 ns MD trajectory was used as input to the ZDOCK server for unbiased docking (without specifying any contacting or repulsion residues) 22. For the top ten docked NCAPH-E2F1 poses, we showed their ZDOCK docking scores in Figure 4D. Poses 1 and 2 are the representative poses and are also having similar protein-protein binding interfaces. Structure were rendered using the ChimeraX software 23,24. Visual representation of top 2 of ZDOCK score for the binding of E2F1 to the NCAPH was provided in the 3D map illustrated in Figure 4E. Furthermore, co-immunoprecipitation (Co-IP) results confirmed that NCAPH interacts with E2F1 in PCa cells (Figure 4F).

To further investigate the regulation of the *E2F1* gene by NCAPH in PCa cells, we assessed NCAPH occupancy on the *E2F1* promoter using ChIP analysis. Our analysis demonstrated that NCAPH binds to the proximal promoter of the *E2F1* gene, covering the region from -2000 to -10 relative to its transcription start site (TSS). Notably, the signal for NCAPH enrichment decreased significantly when cells were treated with siNCAPH (Figure 4G). Collectively, these findings reveal that NCAPH interacts with E2F1 and promotes *E2F1* transcription by binding to the proximal promoter of the *E2F1* gene in PCa cells.

### NCAPH is essential for E2F1 mediated cell cycle transition in PCa cells

**Figure 5:**
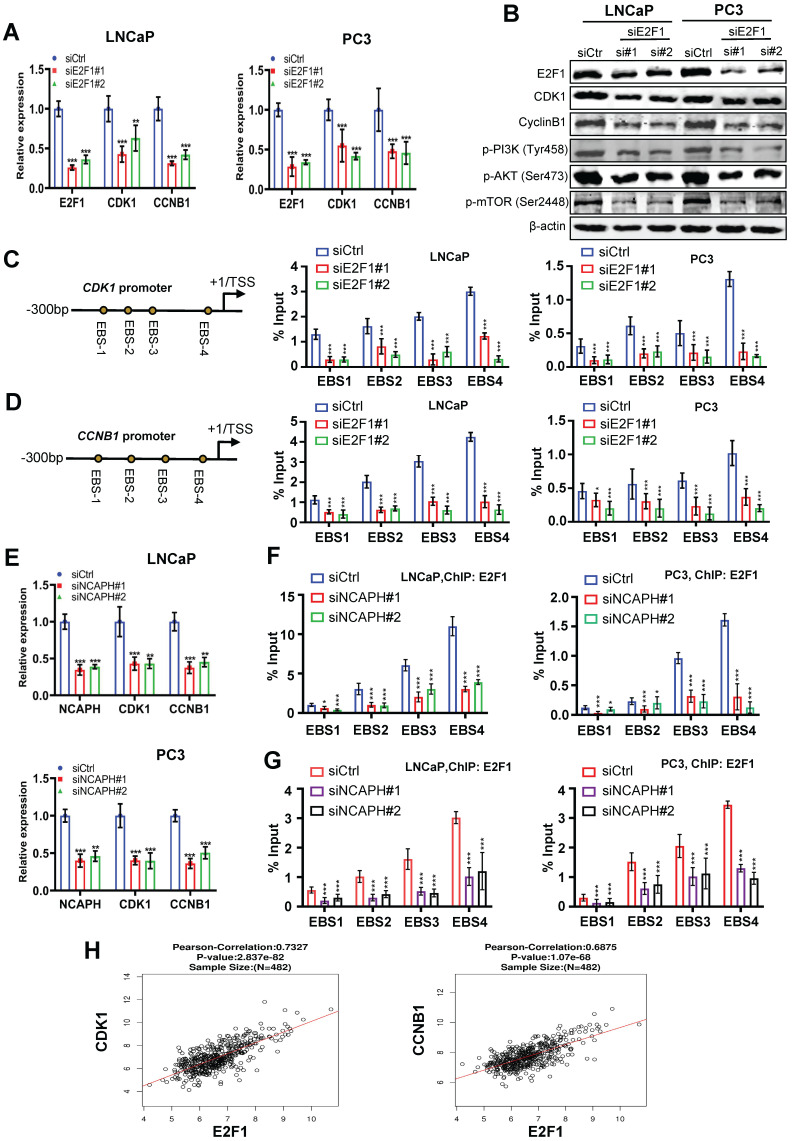
** NCAPH promotes the transcription of* CDK1* and* CCNB1* mediated by E2F1 in PCa cells.** (A) RT-qPCR analysis showing down-regulated *CDK1* and *CCNB1* expression in E2F1-KD LNCaP and PC3 cells. (B) Western blotting analysis showing E2F1-KD caused a strong reduction of CDK1 and Cyclin B1 expression in LNCaP and PC3 cells. (C and D) Schematic representation of the position of ChIP-qPCR primers along the *CDK1* and *CCNB1* promoter. ChIP-qPCR analyzing the E2F1 recruitment to the *CDK1* and *CCNB1* promoter in siE2F1#1- or siE2F1#2-treated LNCaP and PC3 cells. (E) RT-qPCR analysis showing down-regulated *CDK1* and *CCNB1* expression in NCAPH-KD LNCaP and PC3 cells. (F and G) ChIP-qPCR analyzing the E2F1 recruitment to the *CDK1* and* CCNB1* promoter in siNCAPH#1- or siNCAPH#2-treated LNCaP and PC3 cells. (H) The correlation between the *E2F1* gene and *CDK1* and *CCNB1* in PCa based on TCGA database. ns, non-significant, * P < 0.05, ** P < 0.01, *** P < 0.001 vs control.

E2F1 is a key promoter regulating cell cycle transitions in PCa cells 25, therefore, we sought to determine whether NCAPH is involved in the cell cycle transitions controlled by E2F1 in PCa cells. Firstly, we found that E2F1-KD significantly reduced the mRNA expression of cell cycle-related markers such as *CDK1* and *CCNB1* (Figure 5A). In addition, the protein expression of CDK1, Cyclin B1, phosphorylated-PI3K, phosphorylated-AKT or phosphorylated-mTOR was inhibited in LNCaP and PC3 cells treated with E2F1-KD (Figure 5B). These results indicated that E2F1 is a key regulator of cell cycle transitions and PI3K/AKT/mTOR pathway in PCa cells, consistent with previous studies 26,27. We then investigated the mechanism by which E2F1 activates CDK1 and CCNB1 in PCa cells. ChIP-qPCR results showed that E2F1 binds to the proximal promoter of *CDK1* or *CCNB1* genes and subsequently regulates their transcription in LNCaP and PC3 cells (Figures 5C and D). On the other hand, we also demonstrated that NCAPH-KD significantly reduced the mRNA expression of *CDK1* and *CCNB1* (Figures 5E). More interestingly, ChIP-qPCR results showed that NCAPH-KD significantly reduced the binding efficiency of E2F1 to the proximal promoters of *CDK1* and *CCNB1*, thereby reducing the transcription of *CDK1* and* CCNB1* genes in these PCa cells (Figures 5F and G). These results indicate that NCAPH is involved in the regulation of *CDK1* and *CCNB1* gene transcription by E2F1 in PCa cells. Finally, we also observed that the expression of *E2F1* mRNA in PCa clinical samples based on the TCGA dataset was positively correlated with the expression of* CDK1* and *CCNB1* mRNA (Figure 5H). Our data indicate that NCAPH is essential for E2F1 to activate *CDK1* and *CCNB1* transcription in PCa cells.

### NCAPH-KD combined with mTOR inhibitor or CDK inhibitor inhibits the growth of PCa cells *in vitro* and *in vivo*

**Figure 6:**
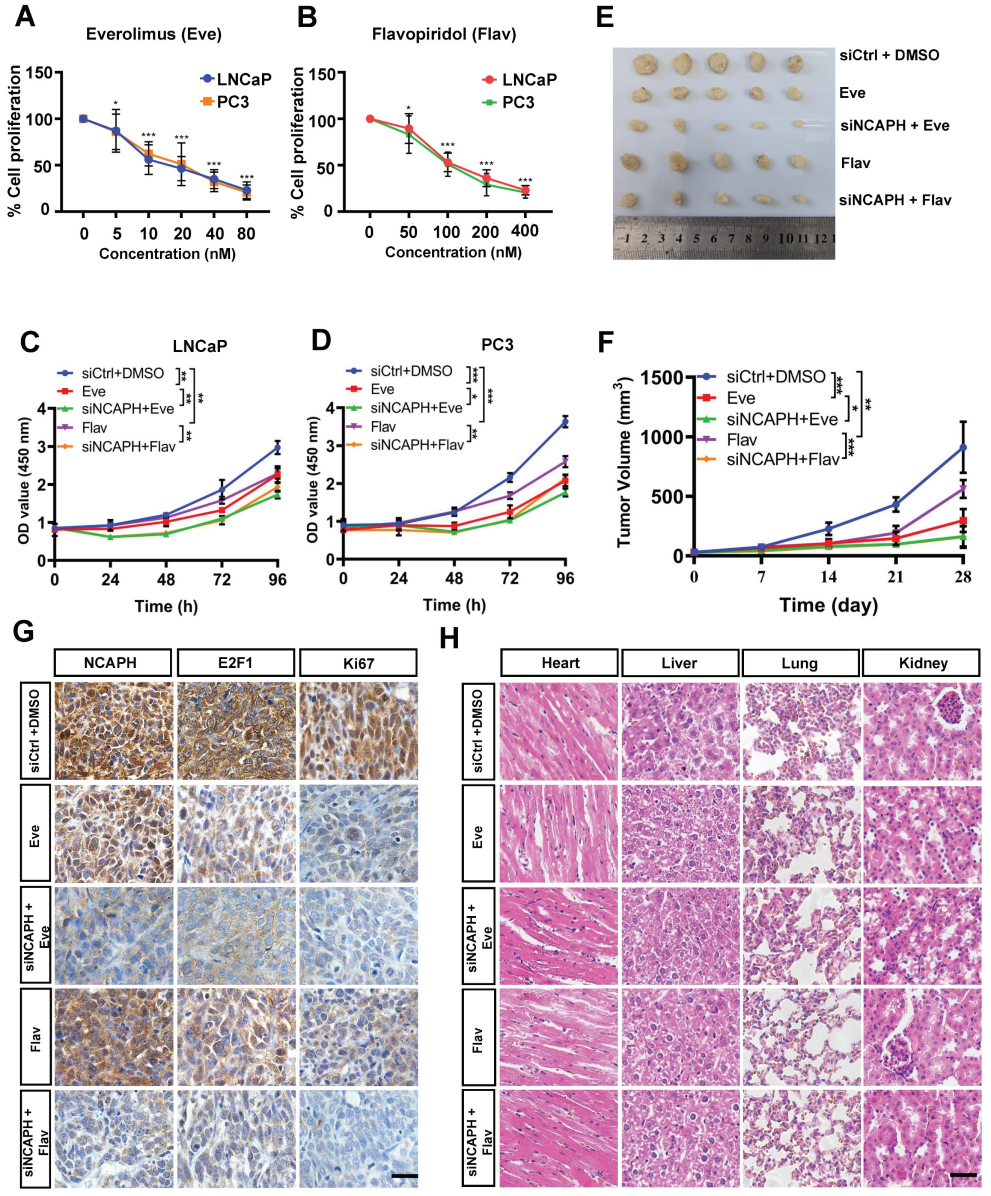
** Combination NCAPH-KD and mTOR inhibitor or CDK inhibitor is an effective strategy for inhibiting the growth of PCa cells.** (A and B) Growth inhibitory effects of different concentrations of Everolimus (Eve) and Flavopiridol (Flav) were determined 48 h after treatment on LNCaP and PC3 cells by CCK-8 assay. (C and D) NCAPH-KD combined with Eve or Flav showed synergistic inhibition on LNCaP and PC3 cells which was determined by CCK8 assay. (E and F) NCAPH-KD combined with Eve or Flav significantly reduced xenografted tumor volume and the xenografted LNCaP cells' growth. (G) Representative IHC staining images for NCAPH, E2F1 and Ki67 in xenografted tumor tissues upon NCAPH-KD combined with Eve or Flav treatment. (H) HE analysis the situation of the heart, liver, kidney and lung in NCAPH-KD combined with Eve or Flav treated-xenografted nude mice. Scale bar, 50 µm. ns, non-significant, * P < 0.05, ** P < 0.01, *** P < 0.001 vs control.

Given our findings that NCAPH promotes the proliferation of PCa cells by activating multiple pathways, such as mTOR and cell cycle transition, we next aimed to investigate the synergistic anti-tumor effects of NCAPH in combination with the mTOR inhibitor Everolimus (Eve) (MedChemExpress, HY-10218) and the cyclin-dependent kinase inhibitor Flavopiridol (Flav) (MedChemExpress, HY-10005). As shown in Figures 6A and B, we observed dose-dependent growth inhibition in LNCaP and PC3 cells treated with either Eve or Flav. These results indicated that both Eve and Flav effectively inhibit the proliferation of PCa cells. Notably, we found that the combination of NCAPH knockdown (NCAPH-KD) with Eve (20 nM) or NCAPH-KD with Flav (100 nM) exhibited significantly stronger anti-proliferative effects on PCa cells compared to the individual treatments (Figures 6C and D). Furthermore, we administered DMSO, Eve, Flav, NCAPH-KD combined with Eve, or NCAPH-KD combined with Flav in a LNCaP cell xenograft nude mouse model. We discovered that NCAPH-KD combined with these two inhibitors significantly reduced the size of xenograft tumors in nude mice compared to the use of either treatment alone (Figures 6E and F). Additionally, we observed that NCAPH-KD combined with these inhibitors resulted in a substantial reduction in the expression of NCAPH, E2F1, and Ki67 in the xenografted tumor tissues when compared to single treatments with Eve or Flav (Figure 6G). Meanwhile, Hematoxylin and Eosin (HE) staining analysis demonstrated that the treatments with NCAPH-KD, Eve, or Flav did not cause any damage to the organs of xenografted nude mice, including the heart, liver, kidney, and lung (Figure 6H). In conclusion, our results indicate that NCAPH-KD can synergistically enhance the anti-proliferative effects of Eve or Flav on PCa cells both *in vitro* and *in vivo*, suggesting that NCAPH is a potential therapeutic target for PCa treatment.

## Discussion

Prostate cancer remains a major contributor to cancer-related mortality in males, particularly in Western countries, with its global incidence on the rise. Despite extensive research efforts, the precise mechanisms underlying the initiation and progression of prostate cancer are not yet fully understood.

Consequently, a thorough investigation into the molecular pathways involved in prostate cancer progression is essential for identifying novel drug targets and therapeutic strategies.

Effective treatment strategies for patients with advanced prostate cancer, including castration-resistant prostate cancer (CRPC) and metastatic castration-resistant prostate cancer (mCRPC), remain elusive because the mechanisms of progression of these diseases are not well understood. Our results suggest that the oncogene *NCAPH* plays an important role in both AR-positive prostate cancer and CRPC cells. NCAPH encodes a member of the Barr protein family that plays a crucial role in the condensin complex. Condensin is an important protein complex responsible for organizing interphase chromatin into chromosomes and promoting their proper segregation during mitosis and meiosis 28. There are two known condensin complexes 29, of which the condensin II complex is mainly located in the nucleus during interphase and binds to chromosomes during mitosis 30. Many studies have shown that increased NCAPH expression levels are associated with the development and prognosis of various types of tumors 31.

While previous research has highlighted the oncogenic role of NCAPH in PCa 14,32, our current study builds upon these findings, revealing a substantial increase in NCAPH levels that correlates positively with disease progression. These results suggest that NCAPH overexpression could serve as a primary oncogenic driver in prostate cancer. However, the exact mechanisms by which NCAPH promotes malignancy in prostate cancer cells remain unclear. Interestingly, data mining analysis indicated associations between NCAPH and E2F1-related pathways, PI3K/AKT/mTORC1 signaling, and other pathways related to cell proliferation, as identified through Gene Set Variation Analysis. Recent studies have demonstrated that NCAPH silencing significantly downregulates the AKT/mTOR signaling pathway, thereby inhibiting proliferation, migration, and invasion in breast cancer cells 33. Our findings revealed that silencing the *NCAPH* gene effectively suppressed proliferation, reduced cell cycle progression in prostate cancer cells by directly inhibiting the PI3K/AKT/mTOR pathway, and decreased the expression of the transcription factor E2F1.

E2F1 is the most classical member of the E2F family and plays a multifaceted role in regulating tumor development by targeting various signaling pathways, including the Wnt/β-catenin pathway 34, the Notch pathway 35, and the PI3K/AKT/mTOR pathway 36. In this study, we demonstrated that knockdown of the *NCAPH* gene resulted in a decrease in both the mRNA and protein levels of E2F1, suggesting that E2F1 may be a potential target of NCAPH in PCa cells. By ChIP analysis, we observed the binding affinity of NCAPH to the proximal promoter of *E2F1* and identified E2F1 as a direct downstream target of NCAPH, mediating its oncogenic function to enhance PI3K/AKT/mTORC1 activity. In addition, our study elucidated for the first time the role of E2F1 as an interacting protein associated with NCAPH in PCa cells. Using molecular docking prediction and Co-IP analysis, we confirmed the interaction between NCAPH and E2F1. The intriguing interaction between NCAPH and E2F1 warrants further investigation of its underlying mechanisms, which will be the focus of our future studies.

Our findings above confirmed that NCAPH promotes cell cycle transitions by activating the PI3K/AKT/mTOR signaling pathway in PCa cells. Therefore, our next goal was to investigate whether silencing the *NCAPH* gene could enhance the sensitivity of PCa cells to PI3K/AKT/mTOR or cyclin-dependent kinase (CDK) inhibitors. We specifically chose to study the mTORC1 inhibitor everolimus (RAD001) 37, which is approved for resensitizing PCa cells to docetaxel 38. In addition, we selected Flavonoids as the first CDK inhibitors to undergo phase I/II clinical trials 39. Flavonoids are pan-cyclin-dependent kinase inhibitors known for their potent efficacy in inducing apoptosis in cancer cells 40. Our data showed that silencing the *NCAPH* gene combined with everolimus or flavonoids significantly inhibits the malignant progression of PCa cells *in vitro* and *in vivo* and is superior to everolimus or flavonoids alone. These results are particularly noteworthy given our limited understanding of the molecular mechanisms of prostate cancer. The identification of NCAPH as a novel tumor promoter represents a promising new therapeutic target.

## Conclusion

**Figure 7:**
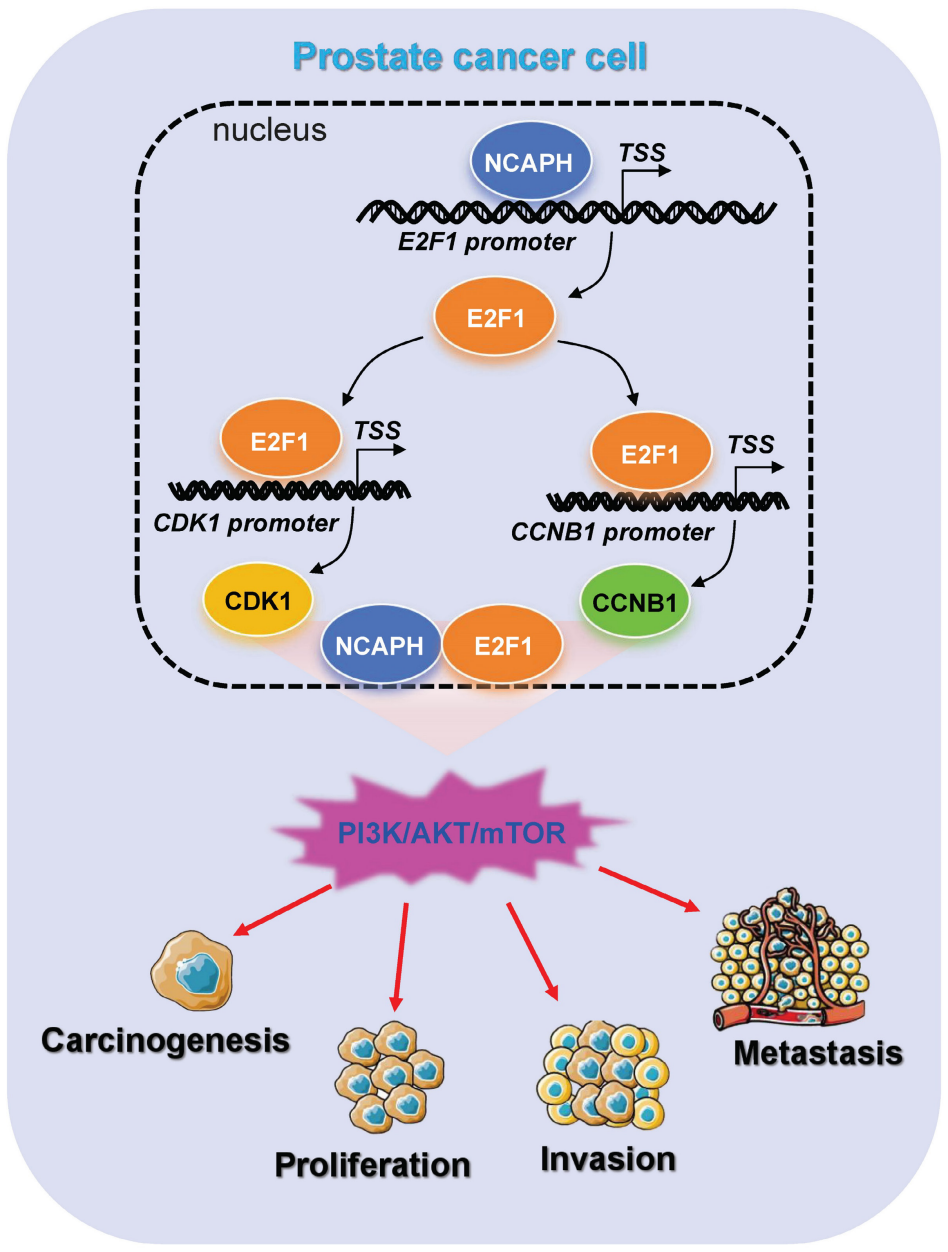
Schematic representation of NCAPH promotes the progression of prostate cancer through activating E2F1 mediated PI3K/AKT/mTOR signaling pathway.

The present study revealed the oncogenic role and regulatory mechanism of NCAPH in the prostate cancer cells (Figure 7). Overall, the results of the present study have provided novel insights into the progression of prostate cancer cells to cancer therapeutics, which may be used to prevent and treat prostate cancer in the future.

## Supplementary Material

Supplementary tables.
