# Supplementary material for: NCAPH Promotes the Proliferation of Prostate Cancer Cells Via Modulating the E2F1 Mediated PI3K/AKT/mTOR Axis
Source: Int J Med Sci. 2025 Jan 27;22(4):940–54. doi: 10.7150/ijms.103444 (PMC11843152; doi:10.7150/ijms.103444)
Supplement: Supplementary file 1 — Supplementary tables. [file ijmsv22p0940s1.pdf]

Supplementary Tables

Supplementary Table S1. Primers used for this study

Purpose	Primers'name	Sequence	Reference
RT-qPCR	NCAPH	F: 5′-GCCAGCCACAATGAATAA-3′	[1]
		R: 5′-TGCAGATCAAAGACCCTC-3′	
	E2F1	F: 5′-GTCACGCTATGAGACCTCAC-3′	[2]
		R: 5′-TCAAGGACGTTGGTGATGTC-3′	
	CDK1	F: 5′-CCTATGGAGTTGTGTATAAGGGT-3′	[3]
		R: 5′-AGCACATCCTGAAGACTGACT-3′	
	CCNB1	F: 5′-CTTGCAGTAAATGATGTGGATG-3′	[4]
		R: 5′-GTGACTTCCCGACCCAGTAG-3′	
	ACTB	F:5′-GCAAAGACCTGTACGCCAACA-3′	[5]
		R: 5′-TGCATCCTGTCGGCAATG-3′	
ChIP-qPCR	BS1 (E2F1 promoter)	F: 5′-GGGTGAGAAGCATCCTCAGGA-3′	
		R: 5′-GGTACTCCAGGTGATGCCAC-3′	
	BS2 (E2F1 promoter)	F: 5′-CAAGTGACCACGTGGTTCTGA-3′	
		R: 5′-GAAAGAAACAGAGGCTAAGCCA-3′	
	BS3 (E2F1 promoter)	F: 5′-AGTAGCTGGGACTACGGGCAC-3′	
		R: 5′-GGCTCACCACCGGAATCCCTG-3′	
	BS4 (E2F1 promoter)	F: 5′-GAATGAGGGAATGAATGAGTT-3′	
		R: 5′-CTGCACCCAGCCCTATCCCGC-3′	
	BS5 (E2F1 promoter)	F: 5′-GTCTCTGGAAATCTAAGCTGAG-3′	
		R: 5′-CCC GCATCCCACTGACCTTTC-3′	
	BS6 (E2F1 promoter)	F: 5′-GCGTTAAAGCCAATAGGAACC-3′	
		R: 5′-TGCCTGCAAAGTCCCGGCCAC-3′	

	EBS-1<br>(CDK1 promoter)	F: 5′-AGGCGGTCTTTTGAGTTTTCC-3′
		R: 5′-CTAATACTTAGGGTTAGGAGTG-3′
	EBS-2<br>(CDK1 promoter)	F: 5′-GTAATGGAATCTCGATGTAAAC-3′
		R: 5′-GCTACTGTTCGCTCCGTTCTTC-3′
	EBS-3<br>(CDK1 promoter)	F: 5′-AGTAGGACGACACTCTCCCGAC-3′
		R: 5′-GAAAGAGGAAAGGGCGGCTAG-3′
	EBS-4<br>(CDK1 promoter)	F: 5′-CTACGGGCTACCCGATTGGTG-3′
		R: 5′-CGCTCTCCGCTCAATTTCCAAG-3′
	EBS-1<br>(CCNB1 promoter)	F: 5′-CAACCCAGAGAGTTGTTG-3′
		R: 5′-GGATCTAGTGTTGCACAC-3′
	EBS-2<br>(CCNB1 promoter)	F: 5′-GACACTTCTGAGACTGTG-3′
		R: 5′-GCCCACCTGGAAAGGTTA-3′
	EBS-3<br>(CCNB1 promoter)	F: 5′-GCTTCACTGCTCTCCAGG-3′
		R: 5′-GGCCACAGTGAGGCTAGG-3′
	EBS-4<br>(CCNB1 promoter)	F: 5′-CCTTCGCGCGATCGCCCTG-3′
		R: 5′-GATTTAAACCCCGCACTG-3′

References:

1. Li B, Xiao Q, Shan L, Song Y. NCAPH promotes cell proliferation and inhibits cell apoptosis of bladder cancer cells through MEK/ERK signaling pathway. Cell Cycle. 2022 Feb;21(4):427-438. doi: 10.1080/15384101.2021.2021050. Epub 2022 Jan 2. PMID: 34974790; PMCID: PMC8855866.

2. Ren Z, Kang W, Wang L, Sun B, Ma J, Zheng C, Sun J, Tian Z, Yang X, Xiao W. E2F1 renders prostate cancer cell resistant to ICAM-1 mediated antitumor immunity by NF-κB modulation. Mol Cancer. 2014 Apr 17;13:84. doi: 10.1186/1476-4598-13-84. PMID: 24742333; PMCID: PMC4004456.Inhibition of CDK1 Overcomes Oxaliplatin Resistance by Regulating ACSL4 - mediated Ferroptosis in Colorectal Cancer.

3. Zeng K, Li W, Wang Y, Zhang Z, Zhang L, Zhang W, Xing Y, Zhou C. Inhibition of CDK1

Overcomes Oxaliplatin Resistance by Regulating ACSL4-mediated Ferroptosis in Colorectal Cancer. Adv Sci (Weinh). 2023 Sep;10(25):e2301088. doi: 10.1002/advs.202301088. Epub 2023 Jul 10. PMID: 37428466; PMCID: PMC10477855.

4. Chen EB, Qin X, Peng K, Li Q, Tang C, Wei YC, Yu S, Gan L, Liu TS. HnRNPR-CCNB1/CENPF axis contributes to gastric cancer proliferation and metastasis. Aging (Albany NY). 2019 Sep 16;11(18):7473-7491. doi: 10.18632/aging.102254. Epub 2019 Sep 16. PMID: 31527303; PMCID: PMC6782008.

5. Jing W, Wang G, Cui Z, Xiong G, Jiang X, Li Y, Li W, Han B, Chen S, Shi B. FGFR3 Destabilizes PD-L1 via NEDD4 to Control T-cell-Mediated Bladder Cancer Immune Surveillance. Cancer Res. 2022 Jan 1;82(1):114-129. doi: 10.1158/0008-5472.CAN-21-2362. Epub 2021 Nov 9. PMID: 34753771.

Supplementary Table S2. Antibodies used for western blotting analysis used in this study.

Name	Supplier	Catalog.	Dilution
anti-NCAPH	Proteintech	11515-1-AP	1:1000
anti-E2F1	Proteintech	66515-1-Ig	1:1000
anti-CDK1	Proteintech	19532-1-AP	1:1000
anti-CyclinB1	Proteintech	55004-1-AP	1:2000
anti-p-AKT	Abcam	Ab38449	1:2500
anti-AKT	Abcam	ab8805	1:2500
anti-p-mTOR	Proteintech	67778-1-Ig	1:1000
anti-mTOR	Proteintech	66888-1-Ig	1:1000
anti-PTEN	Proteintech	22034-1-AP	1:1000
anti-β-actin	Cell Signaling Tech	#4970	1:3000
anti-p-PI3K	Abcam	ab191606	1:2500
anti-PI3K	Abcam	ab302958	1:2500
